# The origins of colour patterns in fossil insects revealed by maturation experiments

**DOI:** 10.1098/rspb.2023.1333

**Published:** 2023-09-20

**Authors:** Shengyu Wang, Maria E. McNamara, Bo Wang, Hejiu Hui, Baoyu Jiang

**Affiliations:** ^1^ State Key Laboratory for Mineral Deposits Research & Lunar and Planetary Science Institute, School of Earth Sciences and Engineering, Nanjing University, Nanjing 210023, Jiangsu, People's Republic of China; ^2^ State Key Laboratory of Palaeobiology and Stratigraphy, Nanjing Institute of Geology and Palaeontology, Chinese Academy of Sciences, 39 East Beijing Road, Nanjing 210008, People's Republic of China; ^3^ School of Biological, Earth & Environmental Sciences, University College Cork, Cork T23 TK30, Ireland; ^4^ Environmental Research Institute, Ellen Hutchins Building, University College Cork, Cork T23 XE10, Ireland

**Keywords:** fossil insect, colour pattern, melanin, taphonomy, insect cuticle, maturation experiments

## Abstract

Many fossil insects show monochromatic colour patterns that may provide valuable insights into ancient insect behaviour and ecology. Whether these patterns reflect original pigmentary coloration is, however, unknown, and their formation mechanism has not been investigated. Here, we performed thermal maturation experiments on extant beetles with melanin-based colour patterns. Scanning electron microscopy reveals that melanin-rich cuticle is more resistant to degradation than melanin-poor cuticle: with progressive maturation, melanin-poor cuticle regions experience preferential thinning and loss, yet melanin-rich cuticle remains. Comparative analysis of fossil insects with monotonal colour patterns confirms that the variations in tone correspond to variations in preserved cuticle thickness. These preserved colour patterns can thus be plausibly explained as melanin-based patterning. Recognition of melanin-based colour patterns in fossil insects opens new avenues for interpreting the evolution of insect coloration and behaviour through deep time.

## 1. Introduction

**Figure 1.**
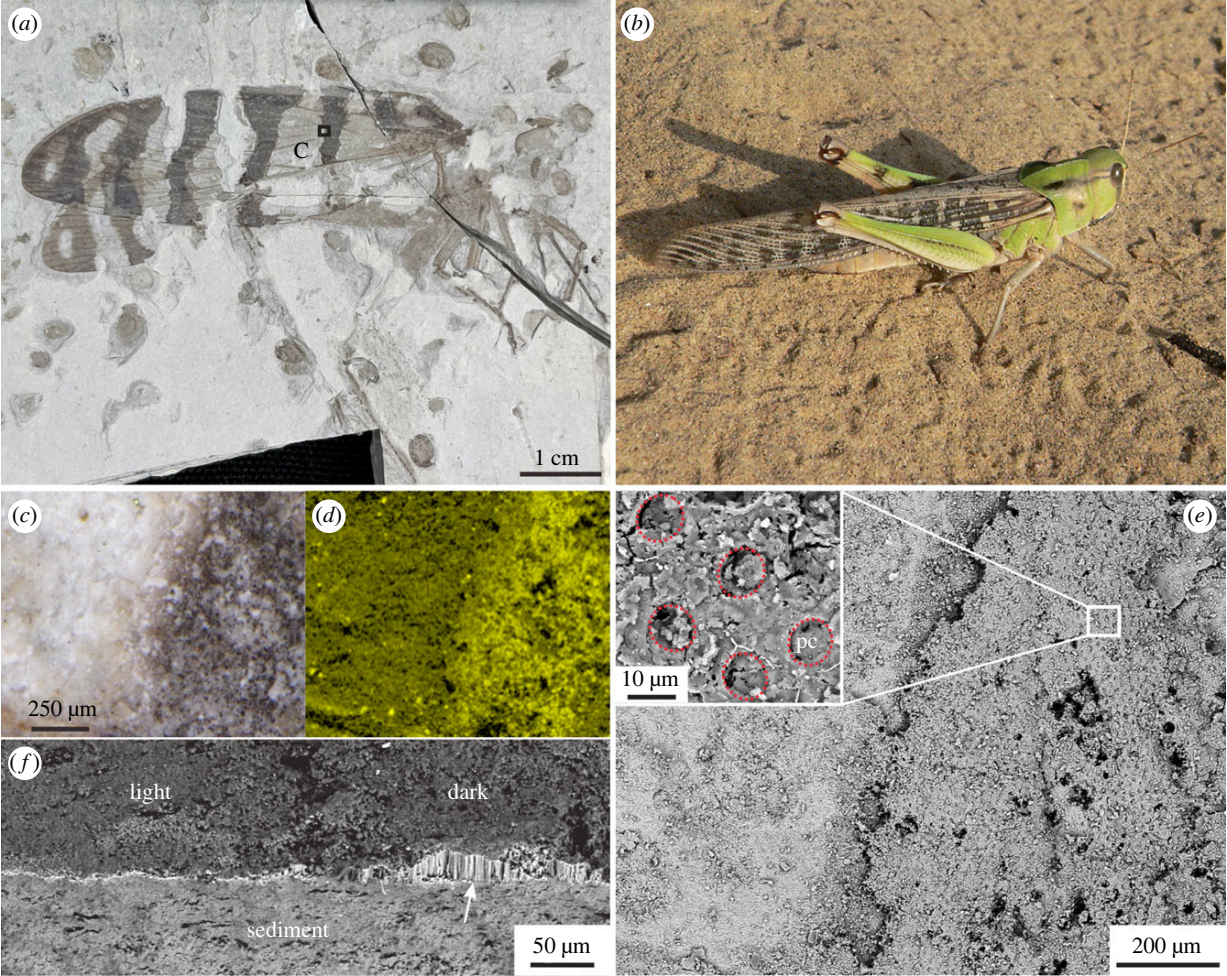
Monochromatic pattern in fossil and extant insects. (*a*) Light micrograph of fossil orthopteran NJU-DHG-01 with alternating dark and light stripes on the wings. (*b*) Photograph of a band-winged locust exhibiting similar wing pattern (courtesy of Christiaan Kooyman). (*c–e*) A close-up light micrograph (*c*), carbon elemental mapping (*d*) and backscattered electron micrograph (*e*) of the region indicated in *a*, the inset in *e* shows regularly arranged pore canals (pc). (*f*) Backscattered electron micrograph of the cross section of fossil showing cuticle (white arrow) preserved only in the dark-toned region.

Insects have been essential components of terrestrial ecosystems since the Early Devonian [1] and often exhibit colour patterns generated by pigments and/or structural colours [2,3]. Such patterns are phylogenetically significant [4] and play important roles in inter- and intra-specific signalling [5]. The evolution of colour patterning in insects, however, remains poorly understood. Monochromatic colour patterns are evident in fossil insects, especially compression fossils, from many Mesozoic and Palaeozoic localities from the Late Carboniferous onwards [6–8]. These tonal patterns appear as spots, bands and blotches that can be brown to black in hue and typically contrast with a lighter-toned background (figure 1). The nature of the fossil patterns has not been investigated; it is plausible that the tonal variation may reflect variations in preserved cuticle thickness, but this has not been tested. The ultimate origin of these fossil patterns also remains speculative, but may reflect variation in sclerotization and/or pigment content [9,10]. The most common pigment in insects is melanin [11], which is distributed diffusely throughout the cuticle [3,12]. Melanin is resistant to degradation [13,14], including thermal maturation [15,16], and evidence of melanin has been detected in cuticles of fossil insects [15,17]. A link between original melanin content and the fossil patterns is therefore plausible, but has not been investigated empirically using taphonomic experiments. The preservational mechanism thus remains unknown.

Here, we test two hypotheses: (i) that the tonal variation in fossil insects reflects variations in cuticle thickness, and (ii) that the thickness of insect cuticles following thermal maturation is influenced by original cuticular melanin content. Our observations on fossil insect cuticle confirm that monochromatic patterns in compression fossils reflect variations in cuticle thickness. Using thermal maturation experiments on extant beetles, we demonstrate that melanin-rich cuticle is more resistant to degradation and melanin-poor cuticle experiences preferential thinning and eventual loss with progressive maturation. Our new data provide important evidence for melanin-based colour patterns in fossil insects.

## 2. Material and methods

### (a) Fossil material

We used an orthopteran specimen (NJU-DHG-01) from the Middle-Late Jurassic Haifanggou Formation of Inner Mongolia, China. The fossil orthopteran has striking monochromatic patterns on the wings (figure 1*a*), which are similar to extant band-winged grasshopper (figure 1*b*). The specimen is now hosted in the School of Earth Sciences and Engineering, Nanjing University.

### (b) Maturation experiments

Our experiments used three extant beetle species: *Pachyteria javana* (Coleoptera: Cerambycidae), *Judolia sexmaculata* (Coleoptera: Cerambycidae) and *Harmonia axyridis* (Coleoptera: Coccinellidae). In each taxon, elytra show colour patterns comprising black spots or blotches on a light-toned background. Melanin has been reported from the cuticle of *Harmonia* [18,19] and is almost certainly responsible for the black coloration in *Judolia* and *Pachyteria*, as black hues in beetle cuticles are usually produced by melanins [20]. The yellow to orange coloration in cerambycids and coccinellids is usually generated by carotenoids [21,22]; ommochromes and flavonoids can also produce yellow-orange hues but have not been reported in beetle elytra [23].

Elytra were wrapped in Al foil and matured in a Genlab MINO/150/DIG oven, a Carbolite CWF1300 furnace, or a Kejing OTF-1200X split horizontal tube furnace (electronic supplementary material, figure S1*a–d*). Experiments were run for 24 h at the following temperatures: 200°C; 250°C; 300°C; 350°C; 400°C; 450°C and 500°C (temperature fluctuated within ± 3°C). At least three replicates were used for each experiment; each replicate used a single elytron. All experiments were carried out in air and at atmospheric pressure.

### (c) Experimental justification

The purpose of the experiments was to assess cuticle thickness in melanin-poor and melanin-rich cuticle regions following maturation at different temperatures. The extant specimens were selected because their simple patterning (comprising large colour blocks) facilitates sampling and correlation of patterning pre- and post-experiment. Temperature was the only variable used here because previous studies have shown that temperature is the primary control on the fidelity of preservation of cuticle structure; pressure has minimal impact [24]. As exceptionally preserved fossils have been inferred to have experienced low, but not necessarily zero, oxygen during their diagenetic history [25], samples were exposed to the natural level of oxygen during the heating process to ensure stable pressure in the furnace. Experimental temperatures were set between 200°C and 500°C because previous experiments have demonstrated retention of cuticle structures at 200°C [24,26], and pilot experiments revealed that gross cuticle morphology was usually lost at 500°C. These experiments also revealed that the cuticle was distorted and fragile following maturation at temperatures greater than or equal to 400°C (electronic supplementary material, figure S1*e–f*), preventing correlation with images of untreated elytra. To promote the recovery of such cuticle fragments, experiments at temperatures greater than or equal to 400°C were repeated using elytra placed flat on commercially available volcanic tephra (Hess pumice, Idaho, USA) in a crucible. The crucible was then wrapped in Al foil with a weight (32–34 g) placed on top to prevent curling and distortion of the elytron during the experiment (electronic supplementary material, figure S1*g–i*). Elytra matured with tephra were used only for the assessment of gross pattern fidelity.

### (d) Light microscopy and scanning electron microscopy

Elytra of the extant beetles were imaged using a Nikon SMZ25 stereomicroscope coupled to a Nikon DS-Ri2 camera before and after each experiment. Untreated elytra and elytra matured at temperatures less than or equal to 350°C were embedded in epoxy resin and polished until the complete longitudinal sections were revealed (electronic supplementary material, figure S2*a–d*); cuticles matured at higher temperatures were too friable to allow embedding in resin. Elytra were platinum- or gold-coated, mounted on Al stubs using carbon tape and examined in high vacuum mode using a JEOL JSM-IT100 variable pressure scanning electron microscope (VP-SEM) at an accelerating voltage of 10 kV and a working distance of 11–20 mm or a Zeiss Sigma 500 SEM at an accelerating voltage of 5 kV and a working distance of 4–8 mm.

Samples (*ca* 10 mm^2^) of fossil cuticle were platinum coated and examined using a Zeiss Sigma 500 SEM equipped with an Oxford X-max 150 energy dispersive spectroscopy (EDS) detector. Observations were made in high vacuum mode at an accelerating voltage of 15 kV and a working distance of 6–8 mm, with acquisition times of 20 min for EDS maps.

### (e) Analysis of cuticle thickness

Beetle elytron comprises a thick elytral dorsal cuticle (EDC) and a thin elytral ventral cuticle (EVC) separated by pillar-like trabeculae [27]. The EDC comprises a thin epicuticle underlain by a thicker exocuticle and, in turn, a thick endocuticle. For *H. axyridis*, EDC epicuticle, exocuticle and endocuticle are 0.2–0.4, 1.9–3.2 and 18.4–23.6 µm thick, respectively. The epicuticle and exocuticle comprise fine laminae (each *ca* 80–200 nm thick); the endocuticle comprises an orthogonal array of bundles of chitin fibres. Cuticular melanin is concentrated in the exocuticle [27,28].

Cuticle thickness was calculated as the sum of the thickness of the EDC and EVC. For each specimen, the complete longitudinal section was imaged (i.e. from the base to the apex) as a mosaic and the images were merged using Adobe Photoshop. Melanin-rich and melanin-poor cuticle regions were located on the SEM images by comparison with microphotographs of untreated elytra (electronic supplementary material, figure S2*e*). Cuticle (and, where possible, exocuticle) thickness was measured from the same location in all specimens matured at temperatures less than or equal to 350°C for each taxon to minimize measurement error, since cuticle thickness varies from the base to the apex of the elytron, especially in *P. javana*.

Differences in cuticle thickness were tested for significance using PAST (PAleontological STatistics v. 4.0) [29] and RStudio for the following sample pairs: (a) melanin-rich and melanin-poor regions of the same elytron; (b) untreated and matured elytra of the same taxon; and (c) elytra (of the same taxon) matured at different temperatures. Note that for (b) and (c), data for melanin-rich and melanin-poor cuticular regions were tested separately. The data for each test pair were tested for normality using the Shapiro–Wilk test. For normal data, differences in variance and mean value between samples were assessed using a two-tailed *F*-test and the *t*-test, respectively. An unequal variance *t*-test, also known as the Welch test, was used in place of the basic *t*-test where variances are significantly different. For non-normal data, a two-tailed Mann–Whitney *U* test was used to compare the medians for each test pair.

## 3. Results

### (a) Fossil colour patterning

The dark-toned regions on the wings preserve cuticle as a continuous carbon-rich layer *ca* 20–30 µm thick that retains surface microstructures, e.g. regularly arranged pore canals (figure 1*e*). Light-toned regions do not preserve cuticle (figure 1*f*) and are depleted in C (figure 1*d*).

### (b) Visible colour patterns

**Figure 2.**
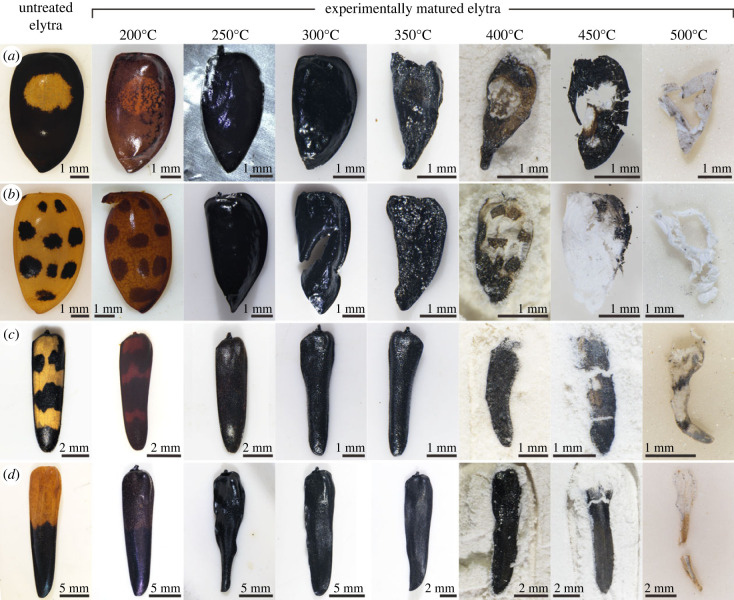
Experimental maturation of insects with melanin-based colour patterns. (*a,b*) *Harmonia axyridis* (Coleoptera: Coccinellidae), (*c*) *Judolia sexmaculata* (Coleoptera: Cerambycidae), (*d*) *Pachyteria javana* (Coleoptera: Cerambycidae).

**Figure 3.**
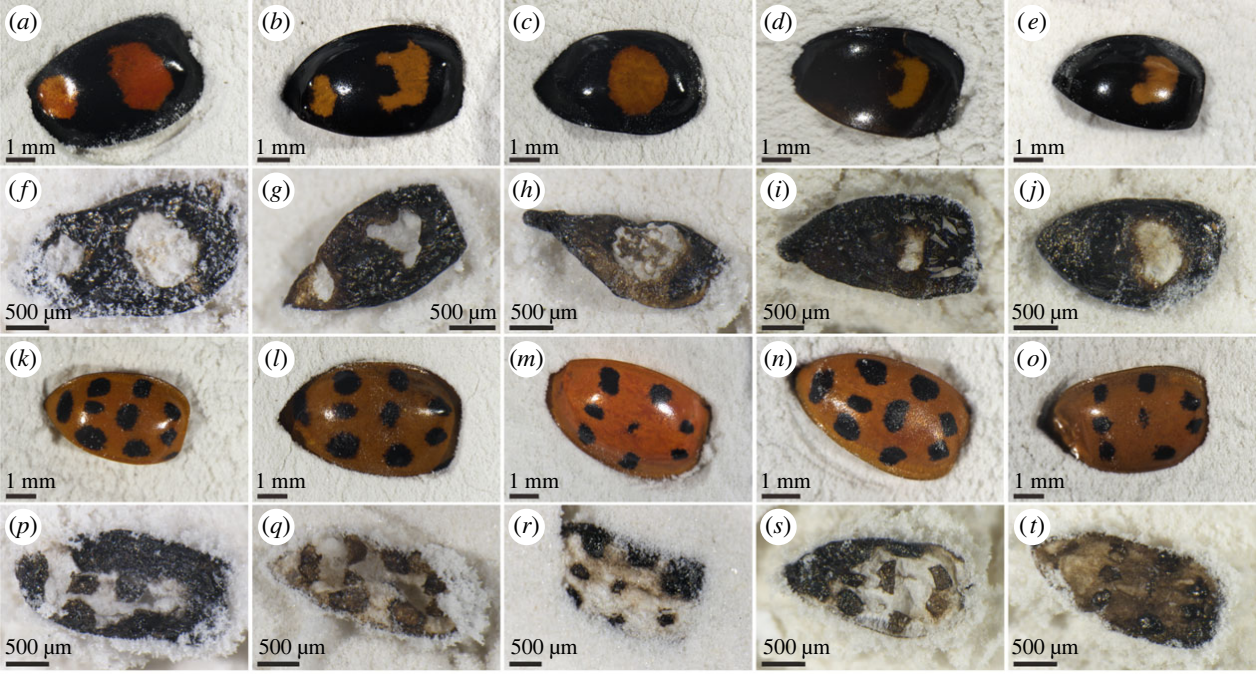
Colour patterning in the elytra of *Harmonia axyridis* (Coleoptera: Coccinellidae) following experimental maturation at 400°C. Panels (*a–e*) and (*k–o*) show untreated specimens of different colour morphs. Panels (*f–j*) and (*p–t*) show the same specimens following maturation.

The visibility of colour patterns differed among experimental treatments. All cuticles matured at 200°C exhibited darkening of melanin-poor regions but retained obvious colour patterning. Following maturation at 250°C, 300°C and 350°C, cuticles transformed to a (near-)uniform black hue, and visible colour patterning was lost in all taxa (figure 2; electronic supplementary material, figure S3). Intriguingly, maturation at higher temperatures resulted in the retention of colour patterns; this phenomenon was apparent for *H. axyridis* following maturation at 400°C and 450°C (figures 2*a,b* and 3) and in *J. sexmaculata* and *P. javana* following maturation at 500°C (figure 2*c,d*)*.* The dark and light regions in the thermally matured colour patterns correspond to originally melanin-rich and melanin-poor cuticle regions, respectively (figure 3); the cuticle in the light-toned regions was very thin or absent*.* Maturation at higher temperatures resulted in the loss of colour patterns and cuticles were transformed to white residues. This phenomenon was apparent for *H. axyridis* (figure 2*a,b*) and can also be observed in some *J. sexmaculata* and *P. javana* (electronic supplementary material, figure S4) following maturation at 500°C.

### (c) Cuticle morphology and ultrastructure

For all taxa, thermal maturation at 200°C did not result in substantial changes in the gross morphology or ultrastructure of elytra (electronic supplementary material, figure S5*b,g,l*). Maturation at temperatures greater than or equal to 250°C, however, resulted in extensive alteration: exocuticular lamination was lost and it was no longer possible to discriminate the exocuticle and endocuticle (electronic supplementary material, figure S5*c,h,m*).

**Figure 4.**
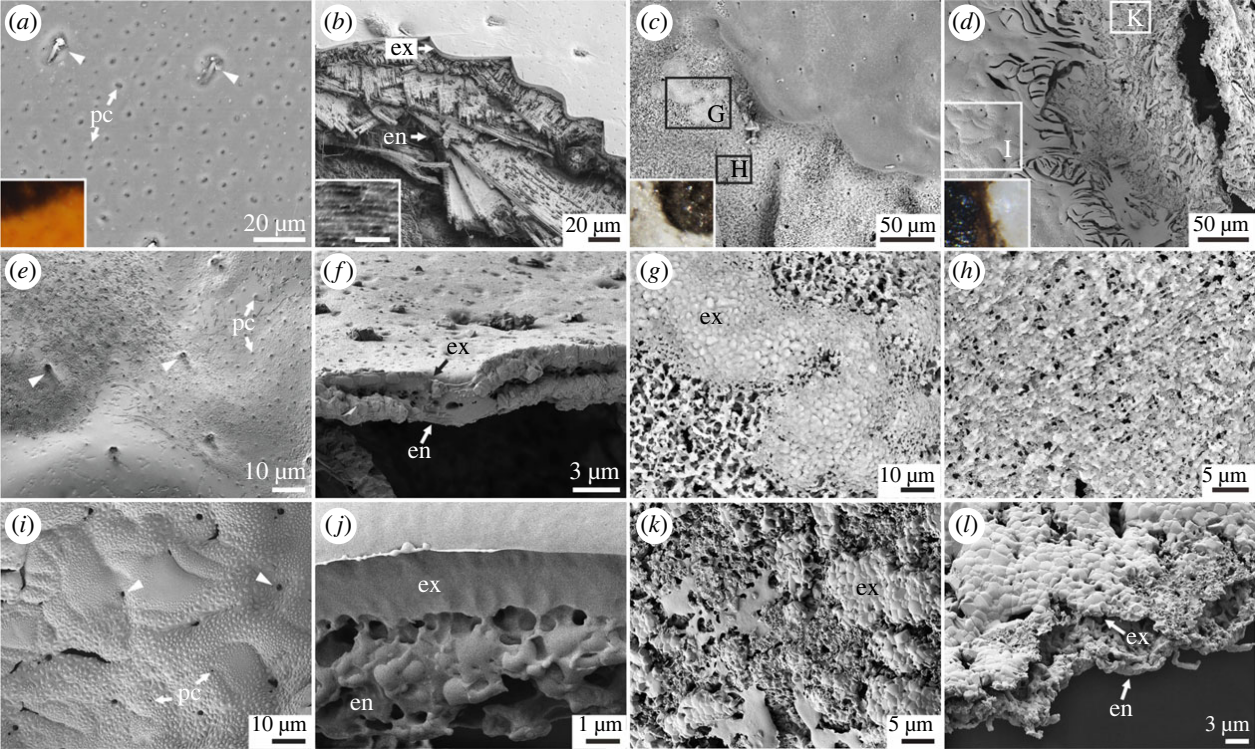
Experimentally induced change in cuticle ultrastructure. (*a–l*) Scanning electron micrographs of untreated and matured cuticle of *Harmonia axyridis* (Coleoptera: Coccinellidae); insets in *a*, *c* and *d* are light micrographs of shown areas*.* (*a,b*) Untreated cuticle; (*a*) Outer surface showing pore canals (pc) and pores of sensilla (arrowhead); (*b*) laminated exocuticle (ex) and endocuticle (en) with fibres overlaying at different angles; inset showing exocuticular lamellae, scale bar: 500 nm. (*c–l*) Cuticle following maturation at 400°C (*c, e–h*) and 450°C (*d, i–l*). (*c, d*) Boundaries of melanin-rich and melanin-poor regions showing discrepancy in degradation; (*e,i*) surface ultrastructure of melanin-rich region showing preserved pore canals and pores of sensilla (arrowhead); (*f,j*) vertical section of melanin-rich region showing a cohesive layer of exocuticle and underlying endocuticular remnant; (*g,k*) cuticular residues in melanin-poor region with no recognizable surface features, exocuticle is degraded to thin patches; (*h*) residue of the endocuticle revealing internal fibre framework; (*l*) vertical section of melanin-poor region showing degraded exocuticle overlying endocuticular residue.

For all taxa matured at 200°C–350°C, there was no difference in cuticle ultrastructure between melanin-rich and melanin-poor regions. At higher temperatures, comparison of melanin-rich and melanin-poor regions was possible only for *H. axyridis* following maturation at 400°C and 450°C (figure 4*c,d*); for other taxa and temperatures, cuticles were either uniform in colour and/or too distorted to allow assessment of the colour pattern. In melanin-rich regions of *H. axyridis*, pores of sensilla and pore canals are evident on the cuticular surface even after maturation at 450°C (figure 4*e,i*). The exocuticle (and possibly epicuticle) is retained as a cohesive surface layer (albeit with loss of internal lamination), and the majority of the endocuticle, except for its outermost layer, is lost (figure 4*f,j*). By contrast, cuticle in melanin-poor regions lacks all recognizable surface features and internal lamination. The cohesive exocuticle is degraded to thin patches (figure 4*g,k*) and underlying endocuticular fibres are revealed (figure 4*h,l*).

### (d) Thickness of cuticle

**Figure 5.**
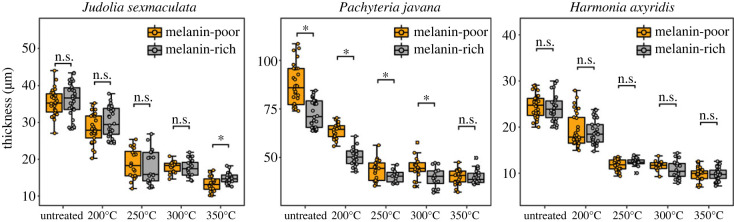
Experimentally induced change in cuticle thickness. Boxplots showing cuticle thickness data following maturation at 200–350°C. Significance levels are indicated above each boxplot: not significant (n.s.); significant (*).

In all taxa, total cuticle thickness and exocuticle thickness both decrease progressively during maturation (figure 5; electronic supplementary material, tables S1 and S2). Most thickness loss is evident following maturation at 250°C, with little additional change following maturation at 300°C and 350°C. Taxa vary, however, in the relationship between cuticle thickness in melanin-rich and melanin-poor regions in untreated and thermally matured elytra. In *J. sexmaculata*, melanin-rich and melanin-poor cuticles are similar in thickness in untreated cuticles and in cuticles matured at temperatures up to 300°C. Following maturation at 350°C, however, melanin-poor cuticle is significantly thinner than melanin-rich cuticle (*p*(same) = 0.0057). In *P. javana*, melanin-poor cuticle is significantly thicker than melanin-rich cuticle in untreated specimens and after maturation at up to 300°C; these differences are not apparent following maturation at 350°C (*p*(same) = 0.7267). Cuticles therefore show preferential thinning in melanin-poor regions in these two taxa. In *H. axyridis*, melanin-rich and melanin-poor cuticle regions have similar thickness in untreated elytra and elytra matured at temperatures up to 350°C.

## 4. Discussion

### (a) Preservation threshold for pigment-based colour pattern

Our experiments reveal that the preservation of melanin-based colour patterns in insect cuticles reflects preferential degradation of melanin-poor cuticle at elevated temperature. The degradation of patterned cuticle is characterized by four stages:

Stage 1: Colour patterns are evident, albeit with alteration of original hues. Surface morphology and internal structures remain intact.
Stage 2: Cuticles are uniformly black; colour patterns are not evident. Cuticles retain surface ultrastructures, but not exocuticle lamination, and are *ca* 50% thinner than untreated cuticles.
Stage 3: Colour patterns reappear due to the progressive preferential thinning and/or loss of melanin-poor cuticle; melanin-rich cuticle (with cuticular ultrastructure) is retained.
Stage 4: Colour patterns are not evident, due to the loss of melanin-poor cuticle and alteration of melanin-rich cuticle to white residues.

Our data also demonstrate inter-taxonomic degradation variation with elevated temperatures. For instance, *H. axyridis* cuticles reach stage 3 at 400°C, but the other taxa do not reach stage 3 until 500°C. This may reflect differences in cuticle thickness: untreated cuticle is thinner in *H. axyridis* (*ca* 20–30 µm) than in the other taxa (*ca* 30–45 µm in *J. sexmaculata* and *ca* 65–105 µm in *P. javana*). Unlike those of other taxa, preferential thinning in melanin-poor regions was not detected in cuticles of *H. axyridis* following maturation until 350°C. It may happen at temperatures higher than 350°C. Significant differences in thickness between melanin-rich and melanin-poor regions are noticeable in *H. axyridis* maturated at 400°C (electronic supplementary material, figure S6). Unfortunately, cuticles matured at 400°C were too friable to allow embedding in resin for the purpose of thickness measuring.

### (b) Broader implications

Our results show that the monochromatic patterns preserved in fossil insects, particularly those in compression fossils, can be attributed to the physical loss of melanin-poor cuticle and thus probably represent the original distribution of melanin-rich and melanin-poor regions of the pattern. This is consistent with chemical evidence for melanin in dark-coloured cuticular residues in other fossil insects [17]. A comparable preservation pattern has been noted in fossil feathers with melanin-based coloration, in which only dark regions of feathers preserve remnents with melanosome films [30]. Maturation experiments have also shown that melanized feathers are more resistant to degradation than non-melanized feathers [31].

Our experimental data suggest that insect cuticles characterized by melanin-based colour patterns follow a convergent degradation pathway during diagenesis. The monochromatic colour patterns can only be apparent under certain taphonomic conditions, which is primarily influenced by diagenetic temperature. Preservation of colour patterns in fossil insects therefore probably reflects relatively mild diagenetic conditions (corresponding to stage 1), or maximum diagenetic temperatures that are intermediate between the respective degradation thresholds of melanin-rich and melanin-poor cuticle (corresponding to stage 3). Intriguingly, our experiments also suggest that uniform dark hues in fossil insects may not always be original, especially where cuticles are well-preserved, i.e. retain internal and surface ultrastructures. In such cases, uniform dark hues may be an artefact of fossilization, reflecting moderately extensive degradation corresponding to stage 2 in our model.

Diagenetic temperature is one of the key factors controlling the extent of diagenetic alteration of colour patterns. Previous studies have investigated the diagenetic alteration of insect structural colour [24,32] and feather colour [33] under elevated temperatures. Our work aims to understand the preservation of pigment-based insect colour patterns, especially for compressed fossils that may have been subjected to elevated temperatures. Colour patterns have been found in various insect biotas with different diagenetic processes, such as these from the Yixian Formation (Cretaceous, China) [34], the Crato Formation (Cretaceous, Brazil) [35], the Green River Formation (Eocene, USA) [36], the Florissant Formation (Eocene, USA) [37] and Kachin amber (Cretaceous, Myanmar) [38]. Further studies are needed to investigate the variations of pattern preservation among different deposits and for different taxa.

Our data represent the first empirical evidence that the monochromatic patterns in compression fossils of insects are biological in origin and represent melanin-based colour patterns. Given that fossil insects representing diverse taxonomic groups and from many biotas exhibit such patterns, our findings have potential applications in the study of the comparative evolution of melanin-based coloration in insects through deep time. Such investigations have the potential to yield valuable insights into the functional evolution of insect coloration, behaviour and physiology in ancient ecosystems.

## Data Availability

The data used in this study are given in the electronic supplementary material [39].
